# Technology‐driven research for radiotherapy innovation

**DOI:** 10.1002/1878-0261.12659

**Published:** 2020-03-19

**Authors:** Claudio Fiorino, Matthias Guckemberger, Marco Schwarz, Uulke A van der Heide, Ben Heijmen

**Affiliations:** ^1^ Medical Physics San Raffaele Scientific Institute Milano Italy; ^2^ Department of Radiation Oncology University Hospital Zurich University of Zurich Switzerland; ^3^ Protontherapy Department Trento Hospital and TIFPA‐INFN Trento Italy; ^4^ Department of Radiation Oncology The Netherlands Cancer Institute Amsterdam The Netherlands; ^5^ Department of Radiation Oncology Leiden University Medical Center Leiden The Netherlands; ^6^ Department of Radiation Oncology Erasmus MC Cancer Institute Rotterdam The Netherlands

**Keywords:** adaptive radiotherapy, innovation, personalized medicine, Radiation Oncology, radiotherapy, research, technology

## Abstract

Technology has a pivotal role in the continuous development of radiotherapy. The long road toward modern ‘high‐tech’ radiation oncology has been studded with discoveries and technological innovations that resulted from the interaction of various disciplines**.** In the last decades, a dramatic technology‐driven revolution has hugely improved the capability of accurately and safely delivering complex‐shaped dose distributions. This has contributed to many clinical improvements, such as the successful management of lung cancer and oligometastatic disease through stereotactic body radiotherapy. Technology‐driven research is an active and lively field with promising potential in several domains, including image guidance, adaptive radiotherapy, integration of artificial intelligence, heavy‐particle therapy, and ‘flash’ ultra‐high dose‐rate radiotherapy. The evolution toward personalized Oncology will deeply influence technology‐driven research, aiming to integrate predictive models and omics analyses into fast and efficient solutions to deliver the best treatment for each single patient. Personalized radiation oncology will need affordable technological solutions for middle‐/low‐income countries, as these are expected to experience the highest increase of cancer incidence and mortality. Moreover, technology solutions for automation of commissioning, quality assurance, safety tests, image segmentation, and plan optimization will be required. Although a large fraction of cancer patients receive radiotherapy, this is certainly not reflected in the worldwide budget for radiotherapy research. Differently from the pharmaceutical companies‐driven research, resources for research in radiotherapy are highly limited to equipment vendors, who can, in turn, initiate a limited number of collaborations with academic research centers. Thus, enhancement of investments in technology‐driven radiotherapy research via public funds, national governments, and the European Union would have a crucial societal impact. It would allow for radiotherapy to further strengthen its role as a highly effective and cost‐efficient cancer treatment modality, and it could facilitate a rapid and equalitarian large‐scale transfer of technology to clinic, with direct impact on patient care.

## Graphical abstract

Technology‐driven research has a pivotal role in the advancement of radiotherapy, still more in the current ‘high‐tech’ era. Larger efforts in developing efficient and affordable technology solutions to deliver personalized radiotherapy, even in middle‐/low‐income countries, are necessary. In order to facilitate a rapid and equalitarian large‐scale transfer of technology to clinic, increased investments in technology‐driven radiotherapy research are highly desirable.

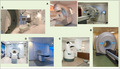

**Abbreviations**
Linac: Linear accelerator
IT: Information technology
3D: three‐dimensional
3DCRT: three‐dimensional conformal radiotherapy
MLC: multileaf collimator
OAR: organ at risk
CT: computed tomography
MRI: magnetic resonance imaging
PET: positron emission tomography
GTV: gross target volume
CTV: clinical target volume
TPS: treatment planning system
IMRT: intensity‐modulated radiotherapy
IMAT: intensity‐modulated arc therapy
VMAT: volumetric‐modulated arc therapy
IGRT: image‐guided radiotherapy
SBRT: stereotactic body radiotherapy
FFF Linac: flattening filter‐free linear accelerator
AI: artificial intelligence
OMD: oligometastatic disease
NSCLC: non‐small‐cell lung cancer
ESMO: European society of medical oncology
GPU: graphical processing unit
NTCP: normal tissue complication probability

## 1 Technology‐driven research in radiotherapy in a historical context

Technology has always had an intrinsic and pivotal role in the development of radiotherapy: Since the early days, technological advancements made the history of radiotherapy. After the discovery of X‐rays and radioactivity, the long road toward modern high‐tech radiation oncology was studded with continuous discoveries, integrating innovative ideas and technology solutions from several disciplines, physics first. The integration of amazing advancements from other disciplines—including mechanical and electronic engineering, computer science, mathematics, imaging physics and technology, statistics, and data sciences—has been crucial for the never‐ending improvement of radiotherapy and the success of radiation oncology (Baumann *et al.*, 2016; Bortfeld and Jeraj, 2011; Thariat *et al.*, 2013). Often, technology developed in other domains was later applied to radiotherapy, such as the Linear accelerator (Linac) technology that replaced 60Co machines during 1960–1980, or the proton and heavy‐ion accelerators, whose development and improvement was much slower and mainly came from the high‐energy and nuclear physics environment (Durante *et al.*, 2017). Again, the rapid advancements in computer sciences permitted the fast development and integration of IT hardware solutions for radiotherapy, as well as of dramatically improved software for treatment plan optimization and delivery.

The efficient and safe translation of new technology from the research domain to the clinical practice has generally been a relatively rapid process in radiotherapy (Baumann *et al.*, 2016): Among the various players, the presence of physicists inside the hospital environment has been a key point of this process (Bortfeld *et al.*, 2015; Fiorino *et al.*, 2015). Medical physics, originally devoted to hospital radiation safety and dosimetry (including the radiotherapy field), has become a pivotal component of the transfer of technology to the clinics and of its testing and refinement. In addition, the feeding link between Hospital/University medical physics and technology developers has always been crucial, although it changes with time and is still changing nowadays.

## 2 The technology revolution of the last two decades: an overview

Over the past 20–25 years, a dramatic technology‐driven revolution has hugely improved the radiotherapy potential for accurate and safe delivery of complex‐shaped dose distributions to the tumor. Similarly to external radiotherapy, research driven by recent technological advances has renewed the potential of the old practice of brachytherapy (through which radioactive sources deliver a radiotherapy dose inside the tumor), as recently reviewed elsewhere (Tanderup *et al.*, 2017).

### 2.1 Conformal radiotherapy

The first step of this revolution was the big jump from a ‘planar imaging‐based’ discipline (i.e., fields were delivered with few open or minimally blocked beams intended to deliver the prescribed dose to very large portions of the body, with ‘high’ probability of including an ‘invisible’ tumor), to the so‐called ‘conformal radiotherapy’ (i.e., the 3D dose distribution is tailored to the target shape). The development and the consequent introduction of the multileaf collimator (MLC) technology during the 1980s and 1990s was a major step toward the potentiation of field shaping according to the shape of tumors. Although the concept of spatially confining the dose strictly around the tumor to spare the surrounding normal tissues (nowadays named ‘Organs at risk’, OARs) was clear since the early days of radiotherapy, it could only materialize after the advent of several technological innovations in imaging, physics, and computer science.

The availability of CTs and, later, of other imaging modalities, including MRI and PET, enabled more accurate identification of targets, namely of both tumors (gross target volume, GTV) and surrounding tissues at risk of micrometastatic spread (clinical target volume, CTV) (ICRU, 2010), as well as their incorporation into the optimization process. Concomitant advances in computer science and dose calculation algorithms opened up the road for individual plan optimization. Again, technology‐driven research and innovations translated into a rapid spread of special computer platforms (the ‘treatment planning systems’, TPS) that can simulate what happens inside the patient when delivering radiation beams, using images as a 3D model of the patient. Unlike their ancestors from the 1970s and 1980s, which were solely used for dose calculation in few representative patient slices (often with a rough approximation), modern TPS are used since the 1990s to optimize shape, position, and weight of multiple beams with the goal of tailoring the dose distribution around the target, while minimizing the dose to OARs. Interestingly, personalized radiotherapy, in the sense of the individual adaptation of the spatial (the geometry) and intensity (the dose) radiation delivery features, started a couple of decades before this concept became familiar to the whole oncology community (Chin *et al.*, 1985). Nowadays, nearly all radiotherapy patients get a treatment that is (highly) personalized on their individual anatomy.

### 2.2 Intensity‐modulated radiotherapy

Another major step for the technological revolution in radiotherapy has been the development of systems for delivering dose distributions strictly tailored to GTV/CTV, thereby extending the ‘conformal radiotherapy’ concept. Modulation of the spatial intensity of the beams in the context of ‘intensity‐modulated radiotherapy’ (IMRT) has hugely increased the degrees of freedom for radiotherapy optimization. Several methods have been implemented to achieve modulation; the most successful and widely used one involves modulation of the 2D intensity of fixed fields through the computer‐controlled motion of MLC systems (Convery and Webb, 1998; Kallman *et al.*, 1988). The extension of this approach led to the so‐called IMAT (intensity‐modulated arc therapy), currently named also as VMAT (‘volumetric’ replacing ‘intensity’), where, instead of delivering the treatment with a number of fixed beams, the radiation is continuously delivered by an X‐ray beam that rotates around the patient (Yu, 1995). This rotational approach further extended the degrees of freedom for optimizing the treatment and, at the same time, made the delivery of complex‐shaped dose distributions more efficient and faster. IMRT/VMAT delivery was accompanied by the rapid development of TPS that included modulation and rotation into the optimization, replacing the traditional trial and error ‘forward’ plan optimization with the so‐called ‘inverse optimization’ (Brahme, 1988), where the theoretically ‘best’ dose distribution was translated into quantitative dose‐volume goals for targets and OARs. Plan optimization of IMRT/VMAT treatments currently resembles a ‘mathematical game’ that determines the optimal ‘physically feasible’ solution for approaching predefined goals.

### 2.3 Image‐guided radiotherapy

Concomitantly to the development and implementation of IMRT technology, technology‐driven research has been largely devoted to the design of in‐room systems for improving the daily patient setup, gradually leading to the clinical use of image‐guided radiotherapy (IGRT) (Jaffray, 2005). Several IGRT approaches have been developed (or are still under development, as shortly described later): planar imaging coupled with the use of internal markers; CT imaging integrated into the Linac (the cone‐beam CT); optical markers; ultrasound‐based systems; and others. These technologies have dramatically improved the accuracy of radiotherapy delivery, and reduced safety margins around GTV/CTV, further enlarging the potential of radiotherapy to spare the healthy tumor‐adjacent tissues. Moreover, many techniques have been developed and implemented to monitor and correct intrafraction changes (including breathing induced motion), so that tumors are effectively tracked during delivery, with a parallel reduction of margins around the target and significantly better sparing of OARs (Korreman, 2012).

**Fig. 1:**
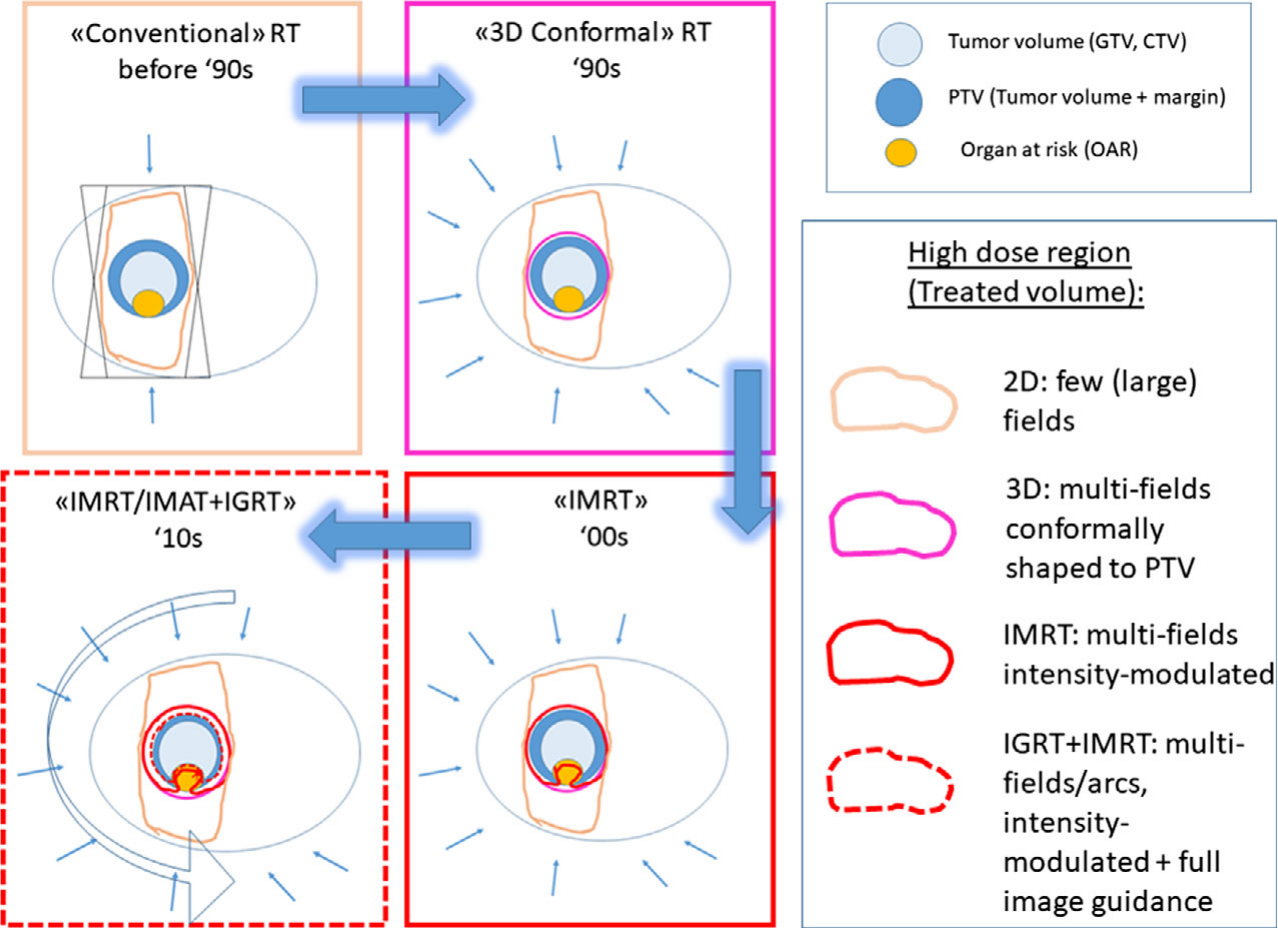
Schematic plot of the impact of technology in the last decades in delivering dose distributions more tailored to GTV/CTV in a typical case of tumor next to an organ at risk: At each step, the high‐dose region corresponding to the previous technologies is overlaid to better appreciate the net benefit. Nowadays, image‐guided intensity‐modulated radiotherapy (using multifields or arc, IMRT, and VMAT, respectively) may strictly tailor the prescribed dose distribution to the tumor, using reduced margins thanks to the high precision of the delivery permitted by IGRT.

In Fig. 1, a schematic cartoon of a concave‐shaped target surrounding a sensitive OAR is used to show how the evolution of technology in the last three decades has improved the ‘conformation’ of the dose distribution around the target, while sparing OARs and adjacent tissues more efficiently.

### 2.4 Extreme hypo‐fractionation and stereotactic body radiotherapy

The widespread implementation of IGRT, mostly in the last decade, and its combination with IMRT/VMAT, largely supported the exploration of extreme hypo‐fractionation. In several relevant situations, as opposed to a conventionally fractionated treatment (with 20–40 fractions), IMRT/VMAT treatment combined with IGRT can be delivered in few fractions (typically 1 to 5), and this has an important impact on both patients and the society, as it is associated with cost reduction and facilitated access to radiotherapy(Atun *et al.*, 2015). This approach represents one of the most valuable successes directly linked to technological innovation and the advancements of IGRT (Jaffray, 2012).

Stereotactic body radiotherapy (SBRT) (Lax *et al.*, 1994) has extended the intracranial radiosurgery concept, contributing to the currently changing clinical paradigm for the treatment of several tumors (Aznar *et al.*, 2018; De Bari *et al.*, 2016; Lewis *et al.*, 2017). The recently developed applications of this stereotactic approach to oligometastatic cancer are described also below.

### 2.5 Recent advancements of delivery technology

In order to make the delivery still more efficient, especially for SBRT applications, flattening‐filter‐free (FFF) Linacs were developed, leading to substantial delivery time reductions (Vassiliev *et al.*, 2006); the replacement of past‐generation machines by FFF Linacs is currently in progress.

The integration of IGRT and IMRT delivery capabilities pushed technology‐driven research to develop improved integrated machines, including ‘special’ dedicated machines that apply various concepts of delivery. Helical tomotherapy (Mackie and Swerdloff, 1993) integrates a 6 MV Linac with a CT, through the helical delivery of an intensity‐modulated continuously rotating fan beam, delivered while the couch translates, similarly to a helical CT scanner. Moreover, the Robotic Linac (Schweikard *et al.*, 2000) treats tumors through the delivery of a large number of ‘pencil’ beams. This machine was also the first example of clinical implementation of tracking: The combination of Robotic Linac with a pair of perpendicular flat panels allows for monitoring patient position during beam delivery and, thanks to a fast feedback technology, offers the possibility to correct beam position in real time if the tumor moves.

## 3 Present and future directions in technology‐driven research

**Fig. 2:**
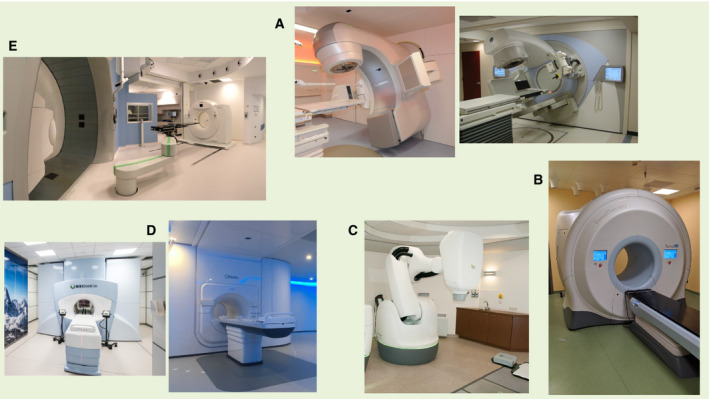
Examples of available ‘High‐Tech image‐guided Linacs’: (A) Conventional Linacs equipped with cone‐beam CT [Zurich UH (left) and NKI‐AvL, Amsterdam (right)]: The kV imaging system is perpendicular to the beam axis and CT images are obtained by rotating the gantry; (B) Helical delivery system (OSR, Milano): The Linac is integrated into a CT ring: Megavoltage images are obtained by using the same treatment fan beam paired to a detector array, delivered in a helicoidal way by moving the couch; (C) Robotic system (Erasmus, Rotterdam): Dose delivery is generated by a large number of small noncoplanar beams delivered by the robotic arms, while image guidance (including tracking during delivery) is driven by a perpendicular pair of flat panel on the floor (in the right corner of the picture) paired to two kV X‐rays tubes positioned on the room ceiling; (D) Hybrid MRI‐Linac machines [Zurich UH (left) and NKI‐AvL, Amsterdam (right)]: The Linac is integrated into an MRI. MRI images can be obtained before and during the treatment delivery. (E) Proton system (Protontherapy, Trento): The gantry (left of the picture) is paired to a robotic couch and a diagnostic CT in the room to setup the patient before treatment delivery

As in part described in the previous section, in modern radiotherapy a range of technologies are in clinical use to treat patients; most of them are highlighted in specific papers of this thematic issue, and Fig. 2 shows in a snapshot most of the available image‐guided equipped technologies actually used in most radiotherapy centers in Europe and North America.

External beam radiotherapy with IMRT/VMAT, particle therapy, and brachytherapy all use different strategies for spatially modulating the deposition of radiation dose in the patient. Nonetheless, they essentially all aim to achieve the same goal: eradicate cancer cells in bulky tumors as well as microscopic regional disease, while at the same time sparing healthy tissue so as to avoid radiation‐induced side effects. This capacity to deposit highly complex‐shaped dose distributions still gives radiotherapy a unique position between the other treatment modalities of cancer: surgery and systemic and targeted drugs. Several themes can be identified in the present and near‐future of technology‐driven research and are here shortly described.

### 3.1 Outlook for technology‐driven research on image guidance

As radiotherapy is noninvasive, the tumor and surrounding anatomy is not visible during the treatment. This is resolved by integrating medical imaging with radiotherapy delivery systems. Cone‐beam CT is widely used in external beam radiotherapy, allowing the therapist to see the patient anatomy at the time of treatment. As previously discussed, this has dramatically increased the precision of radiotherapy and made it possible to increase radiation dose and shortening treatment time while at the same time reducing treatment‐related side effects (Bujold *et al.*, 2012). While CT‐based image guidance has reached a high level of maturity in external beam radiotherapy, the development of suitable image guidance technology is an active research field in heavy particles therapy and in brachytherapy (Landry and Hua, 2018; Tanderup *et al.*, 2017).

A related field of technology‐driven research is still the monitoring and online tracking of tumors, aimed to counteract intrafraction motion, highly relevant in the case of breathing for thoracic and abdominal regions. Much technology‐driven research is oriented to extend the capabilities of fast target recognition as well as of more and more available delivery techniques.

As MRI has superior soft tissue contrast compared to CT, an obvious next step is the integration of MRI systems in image guidance technology. Integrated MRI linear accelerators for external beam radiotherapy are used in tens of institutes worldwide, and this number is increasing rapidly. A key feature of MRI‐guided systems is the capacity to track the cancer not only just prior to irradiation, but while irradiation is ongoing. This allows interrupting the treatment when the tumor moves out of the treatment field, further enhancing precision. Technology for tracking the tumor with the irradiation beams using MRI guidance is currently being developed. Another key feature is the capacity to monitor the response of the tumor to the treatment and to detect changes in tumor characteristics with functional MRI. These techniques are now in development and hold the prospect of further personalizing the treatment to each individual patient (Liney *et al.*, 2018).

### 3.2 Adaptive radiotherapy

The traditional workflow involves an elaborate series of actions to get from diagnostic images to an executable treatment plan. CT, MRI, and PET images need to be registered and tumor volumes and OARs need to be delineated. Treatment plans are designed to optimize radiation beams and intensities to achieve optimal dose distributions. Patients are usually treated with these fixed plans throughout the course of radiotherapy, disregarding treatment response. To accommodate tumor regression and anatomical changes or adjust to the changing biological characteristics of the cancer, a faster feedback loop is required that translates imaging information into a new treatment plan on a much shorter time scale (Sonke *et al.*, 2019).

The concept of adaptive radiotherapy was introduced in 1997 (Yan *et al.*, 1997) and involves modification of the treatment plans at several instances during the course of fractionated radiotherapy. In this way, changes in anatomy such as shrinkage of the tumor can be accommodated. The state of the art involves repeated imaging with cone‐beam CT or diagnostic CT scanner and adaptation of the treatment at a limited number of instances. Another approach (often reported as the ‘plan‐of‐the‐day’) consists of the preparation of plan libraries before starting the treatment, accounting for different anatomical situations. At each fraction, based on the image‐guided tumor visualization, the best plan is chosen, permitting a higher sparing of normal tissues, especially in those situations where CTV coincides with a highly deforming organ, such as bladder or uterus (Thörnqvist *et al.*, 2016).

The technology is however evolving rapidly. Treatment units with CT on rails or integrated MRI‐Linac systems allow for daily online imaging with a high quality. Software for auto‐contouring of images and automatic generation of treatment plans is becoming available commercially, and TPS is becoming sufficiently fast to allow for online adaptation of the treatment during every treatment fraction. A critical step is a validation and clinical approval of the auto‐segmentation and automatically generated treatment plans by radiation oncologists and medical physicists. To reach the goal of online biological image‐guided adaptive radiotherapy, this validation and approval need to be streamlined so that it can be done in a few minutes rather than in hours.

### 3.3 Artificial intelligence and big data

Artificial intelligence (AI) techniques will rapidly find relevant and extensive applications in radiotherapy (Thompson *et al.*, 2018). An important example is the ability of AI in supporting planners to generate automated solutions for treatment planning optimization that are integrating (in part replacing) and improving the traditional, manually optimized, planning (Hussein *et al.*, 2018). AI is also particularly promising to support online treatment planning and adaptive radiotherapy (Boon *et al.*, 2018). The potential for fast reconstruction of CT or MR images has been demonstrated as well as the feasibility to generate CT‐like images that are needed for dose calculations from MRI. Many recent studies show its potential for auto‐contouring of organs at risk and, in some cases, tumors. Using AI for quality assurance and outlier detection may help to speed up the process of validation and clinical approval in an online adaptive workflow (McNutt *et al.*, 2019). Increasingly, traditional AI techniques are replaced by deep learning methods (Meyer *et al.*, 2018; Sahiner *et al.*, 2019). Convolutional neural networks have been shown to be superior in auto‐contouring of OARs, and their application for CT and MRI reconstruction and treatment planning are an active field of research. AI also plays an important role in the development of prediction models for outcome based on the images collected during the course of radiation therapy (Bibault *et al.*, 2016). Radiomics uses the extraction of image features from CT, MRI, and PET to find imaging biomarkers and generate prediction models. It is particularly promising to bridge personalized medicine and radiation oncology (Lambin *et al.*, 2017). As with other AI applications, deep learning is increasingly replacing traditional methods.

To harness the potential of AI, large amounts of data are necessary. Particularly, deep learning algorithms tend to be data hungry. Data sharing is therefore necessary and technology to combine data from different sources in a consistent manner is being developed. IT infrastructures that facilitate data sharing are being constructed. To guarantee patient privacy, distributed learning technology is a promising approach. Much research also dealt with the integration of large data base information into decision‐making support systems toward a fully personalized approach to the treatment (Lambin *et al.*, 2013).

### 3.4 Protons and heavy ions

In the early 2010s, the focus of research and developments was to realize and make available at a large scale the gold standard of beam delivery, that is, pencil beam scanning, which allows to fully take advantage of the possibilities of proton and carbon ion therapy. Currently, big efforts are aimed at improving the imaging tools available for patient positioning and treatment adaptation. When it comes to longer term, research activities in radiotherapy with protons and heavier ions are developing on all fronts (radiobiology, clinic, and technology). Concerning technology, the following main directions can be identified:

a The size and complexity of a proton therapy facility is typically much larger than a conventional radiotherapy installation, creating economical and logistical barriers to the introduction of this new technology. Therefore, there is continuous interest in developing better/cheaper methods for beam production and delivery, via either incremental or radical changes (Schippers et al., 2018).
b Particles heavier than protons are interesting not only for the superior dose distributions, but also because of a differential radiobiological effect between tumor and healthy tissues that can be used to maximize their beneficial effects. Therefore, a few centers are now working to enable treatments with new ion species (e.g., Helium and Oxygen) (Sokol et al., 2017).
c Delivering ‘Flash’ therapy (see next section) with heavy charged particles, protons, in particular is interesting and, in some cases, should be feasible with relatively minor changes to the existing equipment (Patriarca et al., 2018).
d Since proton and heavier charge particles, unlike X‐rays, stop in the patient, it is of course important to develop better (real time) *in vivo* range measurement of therapeutic beams (Xie et al., 2017).

### 3.5 ‘Flash’ therapy and spatial fractionation

A quite new and highly promising field of investigation concerns the so‐called ‘Flash’ radiotherapy, consisting in the delivery of ultra‐high (≥40 Gy·s^−1^) dose‐rate beams (Favaudon *et al.*, 2015). Preclinical studies involving cell lines and animals showed a different response of tumor and normal tissues compared to conventional dose rates normally delivered in radiotherapy (typically ranging between 0.01 and 0.1 Gy·s^−1^), with promisingly high benefit in delivering Flash beams. Much preclinical research is nowadays oriented to investigate the basic phenomenon and the related biology effects in preclinical models. In parallel, technology‐driven research is under development to make more easily available ultra‐high dose rates in clinical Linac and few pioneering examples are available, mostly using electron and proton beams (Bourhis *et al.*, 2019). In the case of positive answers from preclinical research, the clinical implementation of Flash radiotherapy could happen quite quickly with the potential to represent a really new and revolutionary approach. Efficient and safe technology needs to be developed with challenging questions to be faced, like the way the beams can be measured, monitored, and possibly delivered in a multifield arrangement. Of note, the first patient, affected by a highly resistant skin lymphoma, was treated with Flash, with an impressive early result (Bourhis *et al.*, 2020).

Another highly promising field of investigation concerns spatial fractionation, including several methods to create dosimetry micropeaks and valleys within a tumor; despite this approach having a long history, most of its aspects remain to be understood (Billena and Khan, 2019). Technologies to better adapt this approach to clinical applications are currently available (using grids or MLCs) and others are under development, including the generation and delivery of microbeams with photons or protons (Schültke *et al.*, 2017).

## 4 Technology to advance cancer care: the example of SBRT for oligometastatic cancer

Radiotherapy technology has been a key driver in oncology, improving outcome of cancer patients in many fields. Oligometastatic disease (OMD) will be here used as an example of how technology recently (and rapidly) changed the picture in a clinically relevant scenario.

**Fig. 3:**
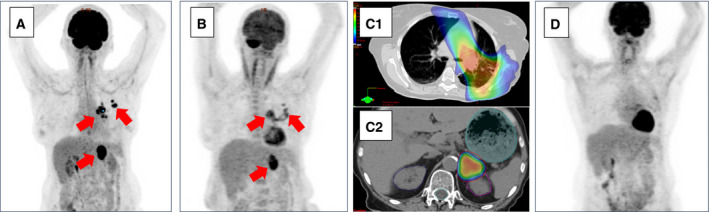
(A) Seventy years, female patient with a diagnosis of synchronous oligometastatic NSCLC: cT2 cN1 cM1b (adrenal), adenocarcinoma, EGFR WT, ALK negative. (B) Status after induction/first‐line chemotherapy. (C) Radical radiotherapy in conventional fractionation for the locoregional primary tumor (C1) and SBRT for the adrenal metastasis (C2). (D) Complete metabolic response 3 months after completion of radiotherapy

OMD is defined as an intermediate cancer state between early stage, where cure is the goal of radical local treatment, and systemic metastasized stage, where local and systemic therapy follows a palliative goal (Hellman and Weichselbaum, 1995). In OMD, cancer has spread beyond locoregional areas, but the metastatic capacity is small such that only few metastases have developed. Radical local treatment of all cancer sites combined with systemic therapy for occult micro‐metastases therefore offers a curative potential. Although the term ‘oligometastases’ was coined and defined in 1995, surgical resection of solitary or limited metastases has been performed for decades and has achieved long‐term disease‐free survival and overall survival for selected patients. However, based on a systematic review of oligometastatic non‐small‐cell lung cancer (NSCLC), surgical resection was almost the exclusive local treatment modality until 2003 and the predominant modality until 2007 (Schanne *et al.*, 2019) with radiotherapy used in only very few patients. This is explained by the inability of ‘traditional’ radiotherapy to locally eradicate oligometastases with sufficient safety and efficacy. Only since the development and broad implementation of SBRT into routine clinical practice, radiotherapy became the local treatment modality with the highest level of evidence and simultaneously the most frequently used local treatment modality in OMD. A patient case example is given in Fig. 3.

Stereotactic body radiotherapy has been developed and first clinically introduced at the Karolinska Hospital in Sweden in 1994 (Lax *et al.*, 1994). SBRT was described and characterized by rigid patient positioning and immobilization in a stereotactic frame, control of breathing‐induced target motion, conformal treatment planning by using noncoplanar treatment techniques, inhomogeneous dose distributions in the target, and dose delivery in few fractions of high single fraction doses. Limitations of SBRT as developed in the 90s and early 2000s included the complex methodology that restricted its use to highly specialized academic centers, as well as the toxicity rates in challenging anatomical situations such as the central lung or upper abdominal region. However, within few years all steps of the SBRT treatment chain were optimized integrating all technologies explained above: Respiratory motion became fully integrated into radiotherapy planning and delivery; dynamic intensity‐modulated radiotherapy combined high conformality with rapid dose delivery minimizing intrafractional uncertainties; and online image guidance ensured accurate treatment delivery.

These technological advances of SBRT and its improved accuracy translated into an improved therapeutic ratio with low risk of toxicity and simultaneously high rates of local tumor control. Safety and efficacy of high‐dose SBRT was demonstrated in metastases of histology previously assumed as radioresistant (Guckenberger *et al.*, 2016; Klement *et al.*, 2018). For pulmonary oligometastases of NSCLC, a matched pair analysis reported the identical outcome of SBRT and surgical metastasectomy and similar promising results of SBRT have been described for other frequent oligometastases locations, such as the liver, adrenal gland, bone metastases, and lymph node metastases. Simultaneously, the technological advances of SBRT not only translated into improved clinical outcome but were also the basis for safe and rapid implementation of SBRT outside of clinical trials and outside of specialized academic centers. An international survey among 1000 radiation oncologists in 2017 reported that 61% of all respondents had implemented SBRT for OMD in clinical routine practice (Lewis *et al.*, 2017). Nevertheless, sufficient institutional experience in SBRT is a prerequisite to ensure optimal outcome (Rieber *et al.*, 2017).

The favorable therapeutic ratio combined with rapid adoption of SBRT was key factor to validate the concept of local ablative treatment for OMD in general. Until today, four randomized controlled trials evaluated the value of local ablative treatment of all macroscopic cancer sites in addition to standard of care systemic therapy. Three randomized controlled trials reported improved overall survival in lung cancer (Gomez *et al.*, 2019), colorectal cancer (Ruers *et al.*, 2017), and a disease agnostic setting (Palma *et al.*, 2019); the fourth study was underpowered for overall survival but reported a significantly improved progression‐free survival (Iyengar *et al.*, 2018): The results regarding NSCLC are summarized in Table 1. Whereas radiofrequency ablation was the exclusive locally ablative treatment modality in the earliest CLOCC trial (Ruers *et al*, 2017), SBRT was the exclusive locally ablative treatment modality in two studies (Iyengar *et al*, 2018; Palma *et al*, 2019) and the most frequent in one study (Gomez *et al*, 2019). Therefore, the current ESMO guideline for oligometastatic NSCLC states that the ‘relative contribution of surgery versus radiotherapy as local treatment modality has not been established yet’ in OMD. Technological progress in the form of SBRT has consequently changed the standard of care from surgery to radiotherapy for the treatment of OMD.

**Table 1:** Summary of the randomized controlled trials comparing standard‐of‐care systemic therapy alone vs systemic therapy plus radical treatment of all cancer lesions for oligometastatic NSCLC (*significant results)

Study	Tumor site	Patients	Radiotherapy as local treatment modality (% of patients)	HR for PFS	HR for OS
Iyengar JAMA Oncol 2018	NSCLC	*N* = 29	100%	0.30*	–
Palma Lancet 2019	Disease agnostic(18% NSCLC)	*N* = 99	100%	0.47*	0.57*
Gomez JCO 2019	NSCLC	*N* = 49	68%	0.30*	0.41*

However, considering the current rapid developments in the field of IT, engineering, and imaging, we expect that radiation technology will remain a key factor in improving the outcome of oligometastatic cancer patients.

Whereas current image guidance achieves accurate targeting of most target lesions, metastases located in the upper abdomen and liver are still challenging for cone‐beam CT‐based or planar X‐ray‐based treatment delivery systems. This may at least partially explain the worse local control rates observed in liver SBRT compared to lung SBRT (Klement *et al.*, 2019). In particular, MRI‐based image guidance solutions are expected to address this challenge, especially when combined with online adaptive radiotherapy (Corradini *et al.*, 2019).

## 5 Challenges and pitfalls of technology‐driven research and of clinical translation

The very strong orientation toward technology‐driven research in radiation oncology has a number of reasons. Although the picture is complex, two general aspects are worthy to be underlined.

First, the process of developing new technology is typically more predictable and with a shorter life cycle than the process of producing and demonstrating improved outcomes. This means that projects focused at generating new devices, or new methods based on software development, are more likely to be successful and their outcome is more easily measurable on a scale of a few years.

Second, the limited dimension and ‘power’ of the radiotherapy community makes it difficult to develop completely new technologies ‘from scratch’. This is the main reason why innovation in the field of radiotherapy‐specific hardware (e.g., new beam delivery systems or radiation detectors) is comparatively slow. However, radiotherapy can greatly benefit from tools and methods initially developed in fields with a much larger ‘critical mass’. Two topical examples are the integration of radiomics (Avanzo *et al.*, 2017) that is benefiting from tools and methods available because of massive investments in the radiology and imaging science domain and, in a more general extent, aimed at developing machine learning in several aspects of life, and the implementation of fast dose calculations and deformable image registration on graphical processing units (GPU) (Pratz and Xing, 2011), which is possible because GPUs were developed in the hugely profitable market of video gaming.

The success of technology in radiation oncology has been so significant that it can actually flip the dynamics between technology and medicine, to the point where it is fair to ask whether technology is driving the future of radiotherapy as opposed to the other way around. Of course, this is not a problem of technology *per se*, but rather an issue that may arise if radiation oncologists and medical physicists are not setting the correct priorities.

In this context, technology‐driven research is prone to issues that roughly belong to three broad categories, as discussed below.

### 5.1 Issues caused by the (implicit or explicit) assumption that technological improvements will inevitably translate into better outcomes, to the point that they do not need clinical validation

A typical example of this approach is to consider planned dose distributions as a very reliable surrogate for clinical outcome. For instance, the transition from 3DCRT to IMRT has been in fact largely justified based on the superiority of IMRT planned dose distributions. Some level of clinical evidence came only after the transition was over (Veldeman *et al.*, 2008). The same argument has been often proposed in the discussions on photon vs proton therapy (Goitein and Cox, 2008).

It is interesting to note, however, that in past few years sophisticated and more quantitative approaches emerged in comparing dose distributions, which may be a good example of how to move forward. Particularly, promising is the so‐called ‘model‐based approach’ (Langendijk *et al.*, 2013) to select patients suitable for a new technology (suggested for instance for proton therapy), which finds a better balance between the possibility of using treatment planning as an outcome predictor and the risk of being overconfident about it. The strength of the model‐based approach is to compare plans based not on pure dosimetric indices but on normal tissue complication probability (NTCP) models taking into account the nonlinear relation between a reduction in dose to the normal tissues and a reduction in radiation side effects. This approach is a good example of how to combine innovative technological approaches (like automatic planning and protons) with the latest knowledge on dose‐effect relations, thus aiming by design at ‘closing the loop’ between technology and clinical outcomes. In addition, even though developed in the context of proton therapy, its domain of applicability is likely to be wider. There are several examples both in the past of radiotherapy (e.g., the early days of IMRT) and in the present (e.g., the MR‐Linac) of situations, where a new technological solution is available, it is theoretically advantageous for a large number of patients, but its availability is scarce, and patient selection is therefore necessary.

### 5.2 Issues caused by prioritizing research primarily according to the appeal of the technological problem

This issue manifests itself in different ways, in particular in medical physics research. There are instances, where significant resources are needed to tackle a problem in its extreme manifestations, while many/most situations can be handled with simpler methods. For example, the problem of treating moving targets in the minority of patients with large respiratory amplitudes is extremely complex, and technologically very interesting. However, for most patients, planning and treatment in free‐breathing may be fully appropriate, as long as systematic positioning errors are minimized (Sonke *et al.*, 2008).

At the other end of the spectrum, there may be research endeavors that are clinically significant but do not receive the attention they deserve because they are not primarily a technological problem. Is the comparatively limited interest in studies of dose‐effect relation caused also by an excessive focus on technology?

### 5.3 Issues related to potentially unmotivated costs increase related to technology

Finally yet importantly, an undesired and totally unintended consequence of emphasizing technological hard problems is to favor the industrial development of radiation oncology devices that are increasingly complex and expensive, potentially taking resources away from developments addressing the need of large and disadvantaged populations, where access to basic radiotherapy facilities is a pressing issue (Lievens *et al.*, 2017).

How can we limit the risk of focusing excessively on technology *per se*? Tighter interaction between research‐oriented and clinic‐oriented professionals is a crucial first step, and there are examples of this process at work in radiation oncology. For decades, proton therapy has been a field where people worked under the assumption that if just the basic scientists made their technology available to clinicians, most, if not all, problems would be solved. This turned out not to be the case. Only much later, when knowledge of nuclear and accelerator physics was brought to the hospitals and integrated with the essential tools of modern radiation oncology, the field took actually off. Similar processes should happen (hopefully faster than in proton therapy) whenever one wants to introduce a high degree of innovation and apply it to a large number of patients. In such cases, neither basic scientists, nor people exclusively involved in clinical practice, can solve the problem by themselves.

## 6 Bridging technology innovation with personalized (and efficient) Radiotherapy

As largely debated in the current thematic issue of *Molecular Oncology*, radiation oncology is a crucial player for the rapid transformation of oncology toward personalized and precision care (Baumann *et al.*, 2016; Krause *et al.*, 2020; Mondini *et al.*, 2020). Among the curative options, it is the only that can merge its increasing ‘surgical’ ability with the potential of sterilizing nodes suspected of infiltration of tumor cells; in addition, the combination with immunotherapy is also expected to extend radiotherapy as a primary player for the ambitious aim of caring (or preventing) metastatic disease (Kroeze *et al.*, 2017). A major element expected to increasingly driving the future is the possibility of more and more using individual (clinical, genetic, other ‘omics’, etc.) information to carefully select patients according to the features of their tumor and to individually predict the risk of toxicity (van der Schaaf *et al.*, 2015). The evolution toward personalization started to influence technology‐driven research and is likely to deeply influence it in the near future, aiming to integrate predictive models into fast and efficient technology solutions for the delivery of the best treatment to single patients. Developments of technology integrating quantitative (morphological and functional) imaging before and during therapy into the optimization and delivery process are expected to be one of the major areas of research of next years. Decision support tools driven by AI‐based platforms integrating large data base information within machine learning systems and data‐sharing communities will also represent a big research item (Lambin *et al.*, 2013), including the selection of the best care and radiotherapy technique. Optimization and delivery are expected to largely benefit of technology solutions permitting a continuously adaptation of the treatment to counteract modifications and incorporating early response into the treatment. In order to efficiently exploit the delivery capabilities, automation will play a key role, pushing technology‐driven research to develop systems that are increasingly able to automatically perform accurate and reproducible patient setup, automatic online planning, and online monitoring of the delivery. The extension of personalized radiation oncology will require affordable technological solutions for high‐, middle‐, and low‐income countries (Lievens *et al.*, 2020), and these last will experience highly increasing cancer incidence and mortality (Atun *et al.*, 2015). Technology solutions for automation of commissioning, quality assurance, safety tests, and planning are likely to play a huge role in technology‐driven research in the field, including new developments in informative science and communication to support remote assistance for therapy personalization.

## 7 Prioritizing technology‐driven radiotherapy research and investments

It is generally accepted that technology‐driven research has been a driver for most major advancements in radiotherapy in the past decades. Most disruptive technologies (3D conformal radiotherapy, intensity‐modulated radiotherapy, cone‐beam CT, MR‐Linac) have been initiated and developed in academic radiotherapy centers and then commercialized by established or startup companies in collaboration with academic researchers. This routing gives some guarantee that commercialized products are indeed innovative and beneficial to cancer patients.

Around 50% of all cancer patients in high‐income countries receive radiotherapy in some stage of their disease. Although exact numbers are unknown, this is certainly not reflected in the worldwide budget for radiotherapy research, as compared, for example, to investments in medical oncology. Pharmaceutical companies spend vast amounts of money on collaborations with academic centers, as drugs can be sold at price levels that allow for substantial investments in innovation. The situation is completely different in radiotherapy research. Resources for research are highly limited in radiotherapy equipment vendors. They generally do not succeed in selling their products at price levels that allow them to substantially invest in innovation. As a result, the investments they can do in collaborations with academic research centers are also rather restricted. This undoubtedly slows down the development of radiotherapy as an effective treatment modality for cancer patients, which is generally more cost‐efficient than medical oncology interventions. The situation may deteriorate further with the current massive introduction of VMAT and hypofractionated treatments, which allow for efficiently treating higher patient numbers with the current treatment machines, thereby putting pressure on the revenues of the radiotherapy industry.

Probably in part caused by the current difficult market situation, more than 90 % X‐rays Linacs are provided by only two companies. Switching equipment vendors is often considered highly undesirable by radiotherapy centers because of the large investments to be made, for example, in training of personnel. Moreover, working with equipment from both vendors is highly costly. This situation does certainly not contribute to substantial investments in research and innovation by the companies.

In a situation as the one sketched above, enhanced investments in technology‐driven radiotherapy research via public funds from national governments, the European Union, and other international organizations (as well as from not‐for‐profit private funds and charities) could have important societal impact. New research investments would probably also result in new startups, thereby improving the current radiotherapy market. More importantly, increased investment in radiotherapy research could facilitate a faster, and equalitarian, large‐scale technology transfer to clinic, with direct impact on patient care, and would consequently enable radiotherapy to further strengthen its role as a highly effective and cheap treatment modality for cancer patients. As technology transfer is slowed down by the high regulatory pressure, as, for example, the one related to the new European Medical Device Regulation, additional investments are needed to effectively counteract this pressure.

## References

[mol212659-bib-0001] Atun R , Jaffray DA , Barton MB , Bray F , Baumann M , Vikram B , Hanna TP , Knaul FM , Lievens Y , Lui TYM *et al* (2015) Expanding global access to radiotherapy. Lancet Oncol 16, 1153–1186. 26419354 10.1016/S1470-2045(15)00222-3

[mol212659-bib-0002] Avanzo M , Stancanello J and El Naqa I (2017) Beyond imaging: The promise of radiomics. Phys Med 38, 122–139. 28595812 10.1016/j.ejmp.2017.05.071

[mol212659-bib-0003] Aznar MC , Warren S , Hoogeman M and Josipovic M (2018) The impact of technology on the changing practice of lung SBRT. Phys Med 47, 129–138. 29331227 10.1016/j.ejmp.2017.12.020 PMC5883320

[mol212659-bib-0004] Baumann M , Mechtild R , Overgaard J , Debus J , Bentzen SM , Daartz J , Richter C , Zips D and Bortfeld T (2016) Radiation Oncology in the era of precision medicine. Nature Cancer Rev 16, 234–249. 10.1038/nrc.2016.18 27009394

[mol212659-bib-0005] Bibault J‐E , Giraud P and Burgun A (2016) Big data and machine learning in radiation oncology: state of the art and future prospects. Cancer Letters 382, 110–117. 27241666 10.1016/j.canlet.2016.05.033

[mol212659-bib-0006] Billena C and Khan AJ (2019) A current review of spatial fractionation: Back to the future? Int J Radiat Oncol Biol Phys 104, 177–187. 30684666 10.1016/j.ijrobp.2019.01.073 PMC7443362

[mol212659-bib-0007] Boon IS , Au Yong TPT and Boon CS (2018) Assessing the role of Artificial Intelligence (AI) in clinical oncology: utility of machine learning in radiotherapy target volume delineation. Medicines (Basel) 5, E131. 30544901 10.3390/medicines5040131 PMC6313566

[mol212659-bib-0008] Bortfeld T and Jeraj R (2011) The physical basis and future of radiation therapy. Br J Radiol 84, 485–498. 21606068 10.1259/bjr/86221320 PMC3473639

[mol212659-bib-0009] Bortfeld T , Torresin A , Fiorino C , Andreo P , Gagliardi G , Jeraj R , Muren LP , Paiusco M , Thwaites D and Knoos T (2015) The research versus clinical service role of medical physics. Radiother Oncol 114, 285–288. 25727681 10.1016/j.radonc.2015.02.003

[mol212659-bib-0010] Bourhis J , Montay‐Gruel P , Gonçalves Jorge P , Bailat C , Petit B , Ollivier J , Jeanneret‐Sozzi W , Ozsahin M , Bochud F , Moeckli R *et al* (2019) Clinical translation of FLASH radiotherapy: Why and how? Radiother Oncol 139, 11–17. 31253466 10.1016/j.radonc.2019.04.008

[mol212659-bib-0011] Bourhis J , Sozzi WJ , Jorge PG , Gaide O , Bailat C , Duclos F , Patin D , Ozsahin M , Bochud F , Germond J‐F *et al* (2020) Treatment of a first patient with FLASH‐radiotherapy. Radiother Oncol 139, 18–22. 10.1016/j.radonc.2019.06.019 31303340

[mol212659-bib-0012] Brahme A (1988) Optimization of stationary and moving beam radiation therapy techniques. Radiother Oncol 12, 129–140. 3406458 10.1016/0167-8140(88)90167-3

[mol212659-bib-0013] Bujold A , Craig T , Jaffray D and Dawson LA (2012) Image‐guided radiotherapy: has it influenced patient outcomes? Semin Radiat Oncol 22, 50–61. 22177878 10.1016/j.semradonc.2011.09.001

[mol212659-bib-0014] Chin LM , Siddon RL , Svensson GK and Rose C (1985) Progress in 3‐D treatment planning for photon beam therapy. Int J Radiat Oncol Biol Phys 11, 2011–2020. 4055457 10.1016/0360-3016(85)90286-x

[mol212659-bib-0015] Convery DJ and Webb S (1998) Generation of discrete beam‐intensity modulation by dynamic multileaf collimation under minimum leaf separation constraints. Phys Med Biol 43, 2521–2538. 9755943 10.1088/0031-9155/43/9/007

[mol212659-bib-0016] Corradini S , Alongi F , Andratschke N , Belka C , Boldrini L , Cellini F , Debus J , Guckenberger M , Hörner‐Rieber J , Lagerwaard F *et al* (2019) MR‐guidance in clinical reality: current treatment challenges and future perspectives. Radiat Oncol 14, 92. 31167658 10.1186/s13014-019-1308-y PMC6551911

[mol212659-bib-0017] De Bari B , Arcangeli S , Ciardo D , Mazzola R , Alongi F , Russi EG , Santoni R , Magrini SM and Jereczek‐Fossa BA (2016) Extreme hypofractionation for early prostate cancer: biology meets technology. Cancer Treat Rev 50, 48–60. 27631875 10.1016/j.ctrv.2016.08.005

[mol212659-bib-0018] Durante M , Orecchia R and Loeffler JS (2017) Charged‐particle therapy in cancer: clinical uses and future perspectives. Nature Rev Clin Oncol 14, 483–495. 28290489 10.1038/nrclinonc.2017.30

[mol212659-bib-0019] Favaudon V , Fouillade C and Vozenin MC (2015) Ultrahigh dose‐rate “flash” irradiation minimizes the side effects of radiotherapy. Cancer Radiother 19, 526–531. 26277238 10.1016/j.canrad.2015.04.006

[mol212659-bib-0020] Fiorino C , Muren LP , Clark CH , van Elmpt W and Jornet N (2015) Expanding the scientific role for medical physics in radiotherapy: time to act. Radiother Oncol 117, 401–402. 26700599 10.1016/j.radonc.2015.11.007

[mol212659-bib-0021] Goitein M and Cox JD (2008) Should randomized clinical trials be required for proton radiotherapy? J Clin Oncol 26, 175–176. 18182658 10.1200/JCO.2007.14.4329

[mol212659-bib-0022] Gomez DR , Tang C , Zhang J , Blumenschein GR , Hernandez M , Lee JJ , Ye R , Palma DA , Louie AV , Camidge DR et al. (2019) Local consolidative therapy Vs. maintenance therapy or observation for patients with oligometastatic non–small‐cell lung cancer: long‐term results of a multi‐institutional, phase II, randomized study. J Clin Oncol 37, 1558–1565. 31067138 10.1200/JCO.19.00201 PMC6599408

[mol212659-bib-0023] Guckenberger M , Klement RJ , Allgäuer M , Andratschke N , Blanck O , Boda‐Heggemann J , Dieckmann K , Duma M , Ernst I , Ganswindt U et al. (2016) Local tumor control probability modeling of primary and secondary lung tumors in stereotactic body radiotherapy. Radiother Oncol 118, 485–491. 26385265 10.1016/j.radonc.2015.09.008

[mol212659-bib-0024] Hellman S and Weichselbaum RR (1995) Oligometastases. J Clin Oncol 13, 8–10. 7799047 10.1200/JCO.1995.13.1.8

[mol212659-bib-0025] Hussein M , Heijmen BJ , Verellen D and Nisbet A (2018) Automation in intensity‐modulated radiotherapy treatment planning – a review of recent innovations. Br J Radiol 91, 20180270. 30074813 10.1259/bjr.20180270 PMC6319857

[mol212659-bib-0026] ICRU Report 83 (2010) *Journal of the ICRU*, Oxford University Press 10 No 1.

[mol212659-bib-0027] Iyengar P , Wardak Z , Gerber DE , Tumati V , Ahn C , Hughes RS , Dowell JE , Cheedella N , Nedzi L , Westover KD *et al* (2018) Consolidative radiotherapy for limited metastatic non–small‐cell lung cancer: a phase 2 randomized clinical trial. JAMA Oncol 4, e173501. 28973074 10.1001/jamaoncol.2017.3501 PMC5833648

[mol212659-bib-0028] Jaffray DA (2005) Emergent technologies for 3‐dimensional image‐guided radiation delivery.Sem. Radiat Oncol 15, 208–216. 10.1016/j.semradonc.2005.01.003 15983946

[mol212659-bib-0029] Jaffray DA (2012) Image‐guided radiotherapy: from current concept to future perspectives. Nature Rev Clin Oncol 9, 688–699. 23165124 10.1038/nrclinonc.2012.194

[mol212659-bib-0030] Kallman P , Lind B , Eklof A and Brahme A (1988) Shaping of arbitrary dose distributions by dynamic multileaf collimation. Phys Med Biol 33, 1291–1300. 3231672 10.1088/0031-9155/33/11/007

[mol212659-bib-0031] Klement RJ , Hoerner‐Rieber J , Adebahr S , Andratschke N , Blanck O , Boda‐Heggemann J , Duma M , Eble M , Eich H , Flentje M *et al* (2018) Stereotactic body radiotherapy (SBRT) for multiple pulmonary oligometastases: analysis of number and timing of repeat SBRT as impact factors on treatment safety and efficacy. Radiother Oncol 127, 246–252. 29510865 10.1016/j.radonc.2018.02.016

[mol212659-bib-0032] Klement RJ , Abbasi‐Senger N , Adebahr S , Alheid H , Allgaeuer M , Becker G , Blanck O , Boda‐Heggemann J , Brunner T , Duma M *et al* (2019) The impact of local control on overall survival after stereotactic body radiotherapy for liver and lung metastases from colorectal cancer: a combined analysis of 388 patients with 500 metastases. BMC Cancer 19, 173. 30808323 10.1186/s12885-019-5362-5 PMC6390357

[mol212659-bib-0033] Korreman SS (2012) Motion in radiotherapy: photon therapy. Phys Med Biol 57, R161–R191. 23165229 10.1088/0031-9155/57/23/R161

[mol212659-bib-0034] Krause M , Alsner J , Linge A , Butof R , Lock S and Bristow R (2020) Specific requirements for translation of biology into clinical radiation oncology. Mol Oncol, (in press). 10.1002/1878-0261.12671 PMC7332213 32175659

[mol212659-bib-0035] Kroeze SGC , Fritz C , Hoyer M , Lo SS , Ricardi U , Sahgal A , Stahel R , Stupp R and Guckenberger M (2017) Toxicity of concurrent stereotactic radiotherapy and targeted therapy or immunotherapy: a systematic review. Cancer Treat Rev 53, 25–37. 28056412 10.1016/j.ctrv.2016.11.013

[mol212659-bib-0036] Lambin P , Leijenaar RTH , Deist TM , Peerlings J , de Jong EEC , van Timmeren J , Sanduleanu S , Larue RTHM , Even AJG , Jochems A *et al* (2017) Radiomics: the bridge between medical imaging and personalized medicine. Nat Rev Clin Oncol 14, 749–762. 28975929 10.1038/nrclinonc.2017.141

[mol212659-bib-0037] Lambin P , Van Stiphout RGPM , Starmans MHW , Rios‐Velazquez E , Nalbantov G , Aerts HJWL , Roelofs E , Van Elmpt W , Boutros PC , Granone P *et al* (2013) Predicting outcomes in radiation oncology‐multifactorial decision support systems. Nature Rev Clin Oncol 10, 27–40. 23165123 10.1038/nrclinonc.2012.196 PMC4555846

[mol212659-bib-0038] Landry H and Hua CH (2018) Current state and future applications of radiological image guidance for particle therapy. Med Phys 45, e1086–e1095. 30421805 10.1002/mp.12744

[mol212659-bib-0039] Langendijk JA , Lambin P , De Ruysscher D , Widder J , Bos M and Verheij M (2013) Selection of patients for radiotherapy with protons aiming at reduction of side effects: the model‐based approach. Radiother Oncol 107, 267–273. 23759662 10.1016/j.radonc.2013.05.007

[mol212659-bib-0040] Lax I , Blomgren H , Näslund I and Svanström R (1994) Stereotactic radiotherapy of malignancies in the abdomen: methodological aspects. Acta Oncol 33, 677–683. 7946448 10.3109/02841869409121782

[mol212659-bib-0041] Lewis S , Porceddu S , Nakamura N , Palma DA , Lo SS , Hoskin P , Moghanaki D , Chmura SJ and Salama JK (2017) Definitive Stereotactic Body Radiotherapy (SBRT) for extracranial oligometastases: an International survey of >1000 radiation oncologists. Am J Clin Oncol 40, 418–422. 25647831 10.1097/COC.0000000000000169

[mol212659-bib-0042] Lievens Y , Borras JM and Grau C (2020) Provision and use of radiotherapy in Europe. Mol Oncol, (in press). 10.1002/1878-0261.12690 PMC7332207 32293084

[mol212659-bib-0043] Lievens Y , Gospodarowicz M , Grover S , Jaffray D , Rodin D , Torode J , Yap ML and Zubizarreta E (2017) Global impact of radiotherapy in oncology: saving one million lives by 2035. Radiother Oncol 125, 175–177. 29173397 10.1016/j.radonc.2017.10.027

[mol212659-bib-0044] Liney GP , Whelan B , Oborn B , Barton M and Keall P (2018) MRI‐Linear accelerator radiotherapy systems. Clin Oncol (R Coll Radiol) 30, 686–691. 30195605 10.1016/j.clon.2018.08.003

[mol212659-bib-0045] Mackie TR and Swerdloff S (1993) Tomotherapy: A new concept for the delivery of dynamic conformal radiotherapy. Med Phys 20, 1709–1719. 8309444 10.1118/1.596958

[mol212659-bib-0046] McNutt TR , Moore KL , Wu B and Wright JL (2019) Use of big data for quality assurance in radiation therapy. Semin Radiat Oncol 29, 326–332. 31472734 10.1016/j.semradonc.2019.05.006

[mol212659-bib-0047] Meyer P , Noblet V , Mazzara C and Lallement A (2018) Survey on deep learning for radiotherapy. Comp Biol Med 98, 126–146. 10.1016/j.compbiomed.2018.05.018 29787940

[mol212659-bib-0048] Mondini M , Levy A , Meziani L , Milliat F and Deutsch E (2020) Radiotherapy‐immunotherapy combinations: perspectives and challenges. Mol Oncol. 10.1002/1878-0261.12658 PMC7332212 32112478

[mol212659-bib-0049] Palma DA , Olson R , Harrow S , Gaede S , Louie AV , Haasbeek C , Mulroy L , Lock M , Rodrigues GB , Yaremko BP *et al* (2019) Stereotactic ablative radiotherapy versus standard of care palliative treatment in patients with oligometastatic cancers (SABR‐COMET): a randomised, phase 2, open‐label trial. The Lancet 393, 2051–2058. 10.1016/S0140-6736(18)32487-5 30982687

[mol212659-bib-0050] Patriarca A , Fouillade C , Auger M , Martin F , Pouzoulet F , Nauraye C , Heinrich S , Favaudon V , Meyroneinc S , Dendale R *et al* (2018) Experimental set‐up for FLASH proton irradiation of small animals using a clinical system. Int J Radiat Oncol Biol Phys 102, 619–626. 30017793 10.1016/j.ijrobp.2018.06.403

[mol212659-bib-0051] Pratz G and Xing L (2011) GPU computing in medical physics: a review. Med Phys 38, 2685–2697. 21776805 10.1118/1.3578605

[mol212659-bib-0052] Rieber J , Abbassi‐Senger N , Adebahr S , Andratschke N , Blanck O , Duma M , Eble MJ , Ernst I , Flentje M , Gerum S , *et al* (2017) Influence of Institutional experience and technological advances on outcome of stereotactic body radiation therapy for oligometastatic lung disease. Int J Radiat Oncol 98, 511–520. 10.1016/j.ijrobp.2016.09.026 27843031

[mol212659-bib-0053] Ruers T , Van Coevorden F , Punt CJA , Pierie J‐PEN , Borel‐Rinkes I , Ledermann JA , Poston G , Bechstein W , Lentz M‐A , Mauer M et al (2017) Local treatment of unresectable colorectal liver metastases: results of a randomized phase II trial. J Natl Cancer Inst 109 10.1093/jnci/djx015 PMC5408999 28376151

[mol212659-bib-0054] Sahiner B , Pezeshk A , Hadjiiski LM , Wang X , Drukker K , Cha KH , Summers RM and Giger ML (2019) Deep learning in medical imaging and radiation therapy. Med Phys 46, e1–e36. 30367497 10.1002/mp.13264 PMC9560030

[mol212659-bib-0055] Schanne DH , Heitmann J , Guckenberger M and Andratschke NHJ (2019) Evolution of treatment strategies for oligometastatic NSCLC patients – a systematic review of the literature. Cancer Treat Rev 80, 101892. 31522079 10.1016/j.ctrv.2019.101892

[mol212659-bib-0056] Schippers JM , Lomax A , Garonna A and Parodi K (2018) Can technological improvements reduce the cost of proton radiation therapy? Semin Radiat Oncol 28, 150–159. 29735191 10.1016/j.semradonc.2017.11.007

[mol212659-bib-0057] Schültke E , Balosso J , Breslin T , Cavaletti G , Djonov V , Esteve F , Grotzer M , Hildebrandt G , Valdman A and Laissue J (2017) Microbeam radiation therapy – grid therapy and beyond: a clinical perspective. Brit J Radiol 90, art no 20170073. 10.1259/bjr.20170073 PMC5853350 28749174

[mol212659-bib-0058] Schweikard A , Glosser G , Bodduluri M , Murphy MJ and Adler JR (2000) Robotic motion compensation for respiratory movement during radiosurgery. Comp Aided Surg 5, 263–277. 10.1002/1097-0150(2000)5:4<263::AID-IGS5>3.0.CO;2-2 11029159

[mol212659-bib-0059] Sokol O , Scifoni E , Tinganelli W , Kraft‐Weyrather W , Wiedemann J , Maier A , Boscolo D , Friedrich T , Brons S , Durante M *et al* (2017) Oxygen beams for therapy: advanced biological treatment planning and experimental verification. Phys Med Biol 62, 7798–7813. 28841579 10.1088/1361-6560/aa88a0

[mol212659-bib-0060] Sonke JJ , Aznar M and Rasch C (2019) Adaptive radiotherapy for anatomical changes. Semin Radiat Oncol 29, 245–257. 31027642 10.1016/j.semradonc.2019.02.007

[mol212659-bib-0061] Sonke JJ , Lebesque JV and Van Herk M (2008) Variability of four‐dimensional computed tomography patient models. Int J Radiat Oncol Biol Phys 70, 590–598. 18037579 10.1016/j.ijrobp.2007.08.067

[mol212659-bib-0062] Tanderup K , Ménard C , Polgar C , Lindegaard JC , Kirisits C and Pötter R (2017) Advancements in brachytherapy. Adv Drug Deliv Rev 109, 15–25. 27637454 10.1016/j.addr.2016.09.002

[mol212659-bib-0063] Thariat J , Hannoun‐Levi J‐M , Sun Myint A , Vuong T and Gérard J‐P (2013) Past, present, and future of radiotherapy for the benefit of patients. Nature Rev Clin Oncol 10, 52–60. 23183635 10.1038/nrclinonc.2012.203

[mol212659-bib-0064] Thompson RF , Valdes G , Fuller CD , Carpenter CM , Morin O , Aneja S , Lindsay WD , Aerts HJWL , Agrimson B , Deville C *et al* (2018) Artificial intelligence in radiation oncology: a specialty‐wide disruptive transformation? Radiother Oncol 129, 421–426. 29907338 10.1016/j.radonc.2018.05.030 PMC9620952

[mol212659-bib-0065] Thörnqvist S , Hysing LB , Tuomikoski L , Vestergaard A , Tanderup K , Muren LP and Heijmen BJM (2016) Adaptive radiotherapy strategies for pelvic tumors – a systematic review of clinical implementations. Acta Oncol 55, 943–958. 27055486 10.3109/0284186X.2016.1156738

[mol212659-bib-0066] Van Der Schaaf A , Langendijk JA , Fiorino C and Rancati T (2015) Embracing phenomenological approaches to normal tissue complication probability modelling: a question of method. Int J Radiat Oncol Biol Phys 91, 468–471. 25680592 10.1016/j.ijrobp.2014.10.017

[mol212659-bib-0067] Vassiliev ON , Titt U , Pönisch F , Kry SF , Mohan R and Gillin MT (2006) Dosimetric properties of photon beams from a flattening filter free clinical accelerator. Phys Med Biol 51, 1907–1917. 16552113 10.1088/0031-9155/51/7/019

[mol212659-bib-0068] Veldeman L , Madani I , Hulstaert F , De Meerleer G , Mareel M and De Neve W (2008) Evidence behind use of intensity‐modulated radiotherapy: a systematic review of comparative clinical studies. Lancet Oncol 9, 367–375. 18374290 10.1016/S1470-2045(08)70098-6

[mol212659-bib-0069] Xie Y , Bentefour EH , Janssens G , Smeets J , Vander Stappen F , Hotoiu L , Yin L , Dolney D , Avery S , O'Grady F *et al* (2017) Prompt gamma imaging for in vivo range verification of pencil beam scanning proton therapy. Int J Radiat Oncol Biol Phys 99, 210–218. 28816148 10.1016/j.ijrobp.2017.04.027

[mol212659-bib-0070] Yan D , Vicini F , Wong J and Martinez A (1997) Adaptive radiation therapy. Phys Med Biol 42, 123–132. 9015813 10.1088/0031-9155/42/1/008

[mol212659-bib-0071] Yu CX (1995) Intensity‐modulated arc therapy with dynamic multileaf collimation: an alternative to tomotherapy. Phys Med Biol 40, 1435–1449. 8532757 10.1088/0031-9155/40/9/004

